# Etiologies of Hospitalized Acute Bronchiolitis in Children 2 Years of Age and Younger: A 3 Years' Study During a *Pertussis* Epidemic

**DOI:** 10.3389/fped.2021.621381

**Published:** 2021-08-12

**Authors:** Sainan Chen, Yuqing Wang, Anrong Li, Wujun Jiang, Qiuyan Xu, Min Wu, Zhengrong Chen, Chuangli Hao, Xunjun Shao, Jun Xu

**Affiliations:** ^1^Department of Respiratory Medicine, Children's Hospital of Soochow University, Suzhou, China; ^2^Department of Pediatrics, Affiliated Suzhou Science and Technology Town Hospital of Nanjing Medical University, Suzhou, China

**Keywords:** *Bordetella pertussis*, bronchiolitis, coinfection, immunization, disease progression, high leukocyte count

## Abstract

**Objective:** In recent years, the incidence of *Bordetella pertussis* infection in infants and young children has been increasing. Multiple studies have suggested that *B. pertussis* may be one of the pathogens of bronchiolitis in infants and young children. However, the prevalence and clinic characteristic of *B. pertussis* in bronchiolitis is controversial. This prospective descriptive study evaluated the prevalence and clinical manifestations of infants and young children hospitalized for bronchiolitis with *B. pertussis*.

**Methods:** Children hospitalized with bronchiolitis were eligible for a prospective study for 36 months from January 1, 2017, to December 31, 2019. Besides *B. pertussis*, 10 common respiratory viruses and *Mycoplasma pneumoniae* (MP) were confirmed by laboratory tests. Medical records of patients were reviewed for demographic, clinical characteristics, and laboratory examination.

**Results:** A total of 1,092 patients with bronchiolitis were admitted. *B. pertussis* was detected in 78/1,092 (7.1%) patients. Of the 78 patients with *B. pertussis* bronchiolitis, coinfections occurred in 45 (57.7%) patients, most frequently with human rhinovirus (28/78, 35.9%), followed by MP (9/78, 11.4%), and human bocavirus (6/78, 7.7%). The peak incidence of *B. pertussis* infection was in May. A high leukocyte count could help distinguish *B. pertussis–*associated acute bronchiolitis from other acute bronchiolitis etiologies. After excluding coinfections, children with *B. pertussis–*only bronchiolitis exhibited a milder clinical presentation than those with RSV-only infection; also, children with MP-only and other pathogen infections revealed similar severity. The morbidity of *B. pertussis* was common (31/78, 39.7%) in infants with bronchiolitis under 3 months.

**Conclusion:** In summary, *B. pertussis* is one of the pathogens in children with bronchiolitis, and coinfection of *B. pertussis* with other viruses is common in bronchiolitis. *B. pertussis* should be considered when patients hospitalized with bronchiolitis present a longer course and have an elevated leukocyte count. Patients with *B. pertussis–*associated bronchiolitis present a milder clinical presentation.

## Introduction

Pertussis, caused by the bacterium *Bordetella pertussis*, is a highly contagious respiratory disease and one of the leading causes of death from infectious diseases in children. *B. pertussis*, a Gram-negative bacterium that was first described by Bordet and Gengou in 1906 (1), has recently reemerged as a major public health threat. The World Health Organization reported 141,074 confirmed pertussis cases worldwide in 2018 (2). Approximately 160,700 deaths were reported worldwide in 2014 from pertussis in children <5 years of age (3).

Bronchiolitis is the most common acute respiratory disease in infants and young children, and one of the most common causes of hospital admission (4, 5). A total of 40–80% of infection is caused by respiratory syncytial virus (RSV), followed by human rhinovirus (HRV), adenovirus (ADV), parainfluenza virus, human bocavirus (hBoV), and human metapneumovirus (hMPV) (6, 7).

In recent years, several studies suggested that *B. pertussis* is a possible pathogen causing bronchiolitis in infants and young children hospitalized for lower respiratory tract infections (8–10). However, studies reporting the prevalence and clinical characteristics of *B. pertussis* bronchiolitis are rare. This study aimed to assess the epidemiological features and clinical characteristics of *B. pertussis* infection and evaluate its impact on infants and young children hospitalized with acute bronchiolitis.

## Materials and Methods

### Patients and Definitions

This prospective descriptive study was conducted on children presenting with acute bronchiolitis who were admitted to the Department of Respiratory Medicine in the Children's Hospital of Soochow University between January 1, 2017, and December 31, 2019. Acute bronchiolitis was characterized by age ≤2, cough, tachypnea, retraction, and expiratory wheezes, often accompanied by rales (11). *B. pertussis* was confirmed by polymerase chain reaction (PCR) assays (12). Patients requiring oxygen supply were considered with severe conditions. The exclusion criteria were as follows: (1) patients with incomplete clinical data; (2) patients with bronchopulmonary dysplasia, heredity metabolic diseases, neurological disorders, congenital heart disease, and immunodeficiency; and (3) patients with evidence suggesting that wheezing was caused by tuberculosis and non-infectious factors such as bronchial foreign bodies.

The study was approved by the ethics committees of Children's Hospital Soochow University (Approval No. 2016026). Informed consent was obtained from the parents of all children enrolled in this study.

### Determination of Vaccination Status

Vaccination history was obtained by querying the “Suzhou Children's Vaccination Inquiry and Evaluation Platform.” A diphtheria, tetanus, and acellular pertussis combination vaccine was administered as a primary series at 3, 4, and 5 months, followed by a booster dose at 24 months in China. The vaccination status was regarded as ever-vaccinated if one to three doses were received.

### Data Collection

Data regarding demographic, clinical, and laboratory characteristics were documented. Demographic and clinical characteristics included age, gender, length of hospital stay, and requirement of supplemental oxygen. Laboratory specimens were obtained including blood and nasopharyngeal aspirates (NPAs). NPAs were obtained during the first 24 h of hospitalization, using a sterile plastic catheter briefly inserted into the lower pharynx *via* the nasal cavity. The blood samples were taken immediately after hospitalization. The laboratory data of leukocyte count, percentages of lymphocytes and neutrophils, and detection of common viruses were collected.

### PCR Detection of *B. pertussis*

*B. pertussis* DNA was detected in NPAs by real-time PCR assays. The primer sequence was synthesized by Shanghai Sangon Biotech Company. The pertussis PtxA-pr and IS481 gene sequences were used as specific primers (Table 1). The RT-PCR assay result was considered negative if the cycle threshold (CT) was ≥40. Specimens that tested positive by PCR for both insertion sequence IS481 (CT < 40) and ptxS1 (CT < 40) were considered positive for *B. pertussis*. If a specimen was PtxA-pr target negative with an IS481 assay CT < 35, it was also considered positive for *B. pertussis*.

**Table 1:** Gene primer sequence and product length detected by real-time PCR.

**Gene name**	**Primer sequence products**	**Length**
IS481	5′GATTCAATAGGTTGTATGCATGGTT3′	145
	5′TGGACCATTTCGAGTCGACG3′	
PtxA-pr	5′CCAACGCGCATGCGTGCAGATTCGTC3′	191
	5′CCCTCTGCGTTTGATGGTGCCTATTTTA3′	

### Respiratory Pathogens

Direct immunofluorescence was used to detect RSV; ADV; influenza virus A (IV-A) and B (IV-B); and parainfluenza virus 1 (PIV I), 2 (PIV II), and 3 (PIV III) using a D^3^ Ultra Respiratory Virus Screening and LD Kit (Diagnostic Hybrids, Athens, OH, USA). A positive result was defined as over five inclusion bodies analyzed under a fluorescence microscope. *Mycoplasma pneumoniae* (MP), HRV, HMPV, and HBoV were detected by a PCR (nucleic acid amplification fluorescent reagent kit, Ann Gene Co., Guangdong, China) according to the manufacturer's instructions.

### Statistical Analyses

Statistical analyses were conducted using SPSS 26.0 (IBM, SPSS, Chicago, IL, USA).

Data were shown as mean ± standard deviation and median and interquartile range. Quantitative variables among the three age groups were compared using one-way analysis of variance or the Kruskal–Wallis test when appropriate. Frequency distribution was compared by the chi-square test. A *p* value <0.05 was considered as a significant difference.

## Results

### Demographic Characteristics

**Figure 1:**
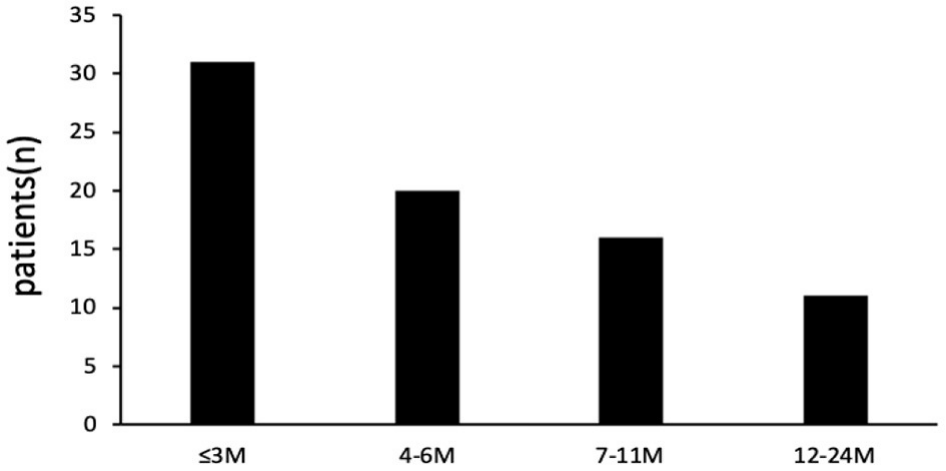
Distribution of *B. pertussis* infection in infants and young children stratified by age.

Of the total 1,092 patients admitted for bronchiolitis, one or more respiratory pathogens including virus and MP were detected in 1,057 of 1,092 patients (a positive rate of 96.8%) and *B. pertussis* was identified in 78 patients (7.1%, based on positive results by PCR). Of the 78 cases of bronchiolitis with *B. pertussis* infection, 47 (60.3%) were male and 31 (39.7%) were female. The male-to-female ratio was 1.52:1. The median age was 6.45 ± 4.94 months. The age distribution of patients is shown in Figure 1; 31 (39.7%) patients were aged ≤3 months, 20 (25.6%) patients were aged 4–6 months, 16 (20.5%) patients were aged 7–11 months, and 11 (14.1%) patients were aged ≥12 months.

### Seasonality of *B. pertussis* Infection

**Figure 2:**
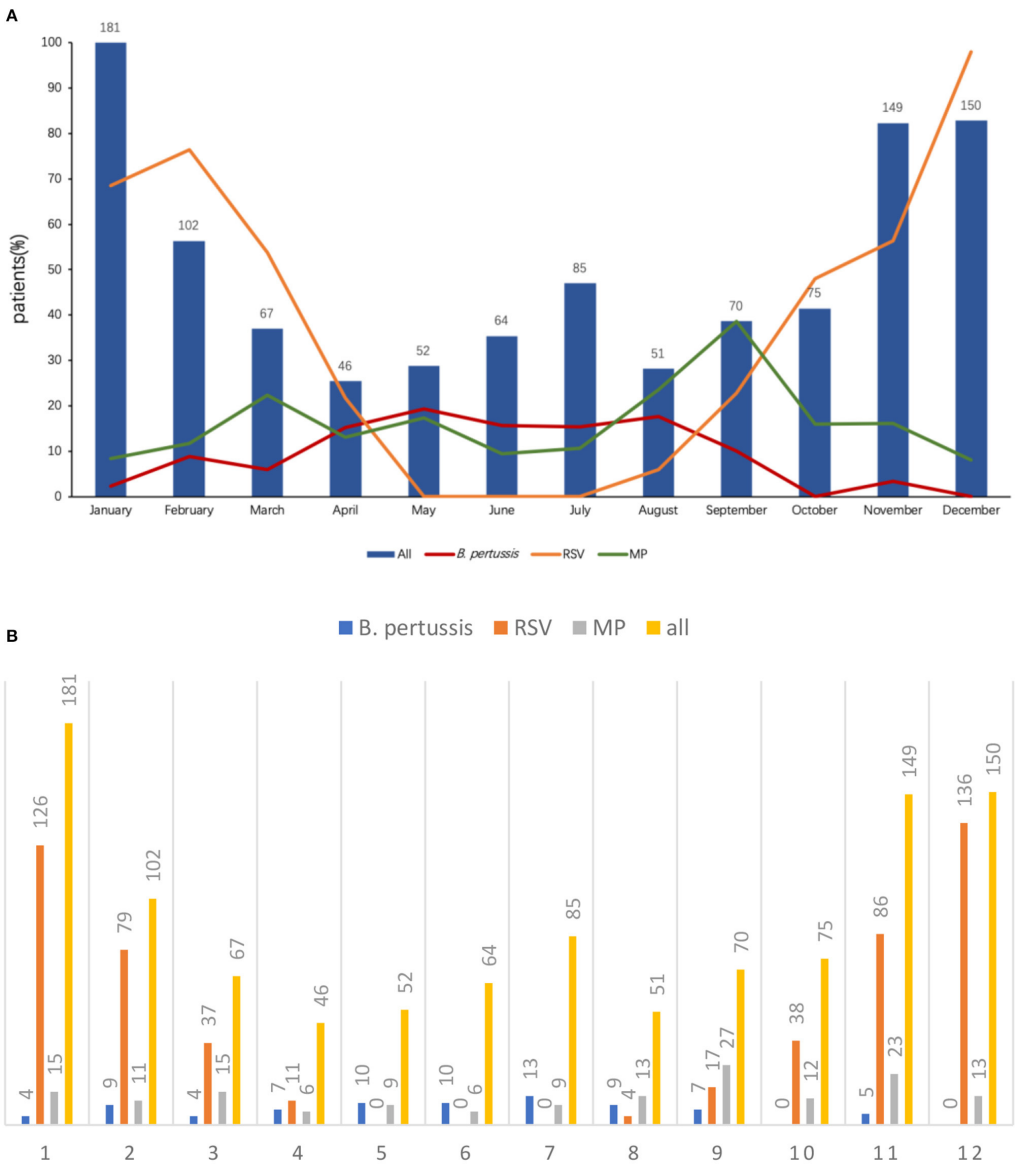
**(A)** Distribution of *B. pertussis* and other pathogen infection by month. **(B)** Distribution of *B. pertussis* and other pathogen infection by month.

The monthly distribution of *B. pertussis* infection is shown in Figures 2A,B. Bronchiolitis could occur throughout the year, and the peak incidence was in winter. The most common pathogen of bronchiolitis was RSV (534/1092, 48.9%), and the peak incidence was in December. MP was detected in 159/1,092 (14.6%) children with bronchiolitis, and the peak incidence was in September. Differing from the above two pathogens, the peak incidence of *B. pertussis* infection was in May, with a total of 10 (19.2%, 10/52) patients reported, and no patients were infected in October and December.

### Coinfection Status

Overall, one or more respiratory pathogens including virus and MP were detected in 1,057 of 1,092 patients. The most commonly detected pathogens in patients with bronchiolitis were as follows: RSV (48.9%), HRV (25.9%), HMPV (13.0%), MP (14.6%), HBoV (12.1%), *B. pertussis* (7.1%), PIV III (7.0%), ADV (1.1%), and PIV I (1.1%).

**Figure 3:**
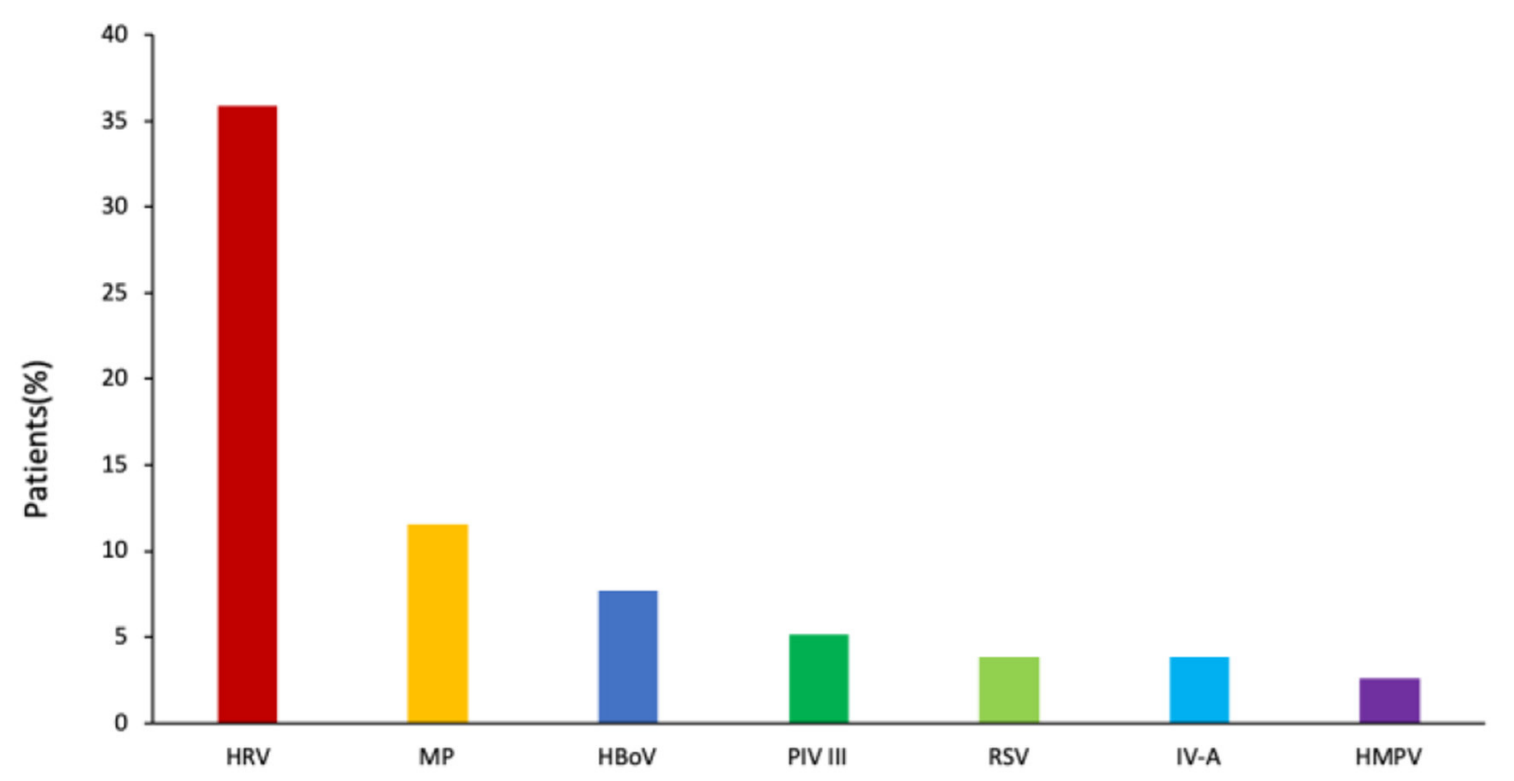
Distribution of pathogen coinfections associated with *B. pertussis* infection.

Of the 78 *B. pertussis–*infected patients, *B. pertussis* was the sole pathogen detected in 33 (42.3%) patients. The remaining 45 patients (57.7%) were coinfected with other respiratory pathogens, most frequently with HRV (*n* = 28, 35.9%), followed by MP (*n* = 9, 11.4%), HBoV (*n* = 6, 7.7%), PIV III (*n* = 4, 5.1%), RSV (*n* = 3, 3.9%), IV-A (*n* = 3, 3.9%), and HMPV (*n* = 2, 2.6%) (Figure 3).

### Clinical Features of *B. pertussis–*Only Infection Compared With Infections With Other Pathogens

In the present study, 33 patients with *B. pertussis*–only infection, 438 patients with RSV-only infection, 87 patients with MP-only infection, and 534 patients infected with other pathogens were analyzed. In unadjusted comparisons, children with *B. pertussis*–only infection were similar to children with RSV-only infection in age, but the number of children with age ≤3 months who were only infected with pertussis was less than that of children with RSV-only infection (Table 2). Children with *B. pertussis*–only infection were significantly more likely to have vomiting (36.4%), cyanosis (12.1%), leukocyte count >15 × 10^9^ (57.6%), longer duration of symptoms before admission (media day, 14.0), and longer hospital stay (media day, 9.0) compared with those with RSV-only infection (17.8%, 2.7%, 7.5%, media 5.0, and 8.0 days, respectively; *p* < 0.05 for all comparisons). Patients with *B. pertussis*–only infection requiring supplement oxygen were fewer than patients with RSV-only infection (6.1 vs. 34.9%; *p* < 0.05).

**Table 2:** Select characteristics of hospitalized children with *B. pertussis-*only infection compared with RSV*-*only, MP*-*only, and other pathogens infection (*n* = 1,092).

	***B. pertussis*–only infection (*n* = 33)**	**RSV-only infection (*n* = 438)**	***MP*-only infection (*n* = 87)**	**Other pathogens infection (*n* = 534)**	***p*-value**
Gender (male/female)	21/12	321/117	54/33	374/160	0.148
Median age, months	3.9 (2.5, 5.0)	2.8 (2.0, 4.9)	5.8 (3.0, 13.0)^**^	6.8 (3.7, 12.0)^*^	**<0.001**
Age group					
≤3 months	17 (51.5%)	300 (68.5%)^**^	30 (34.5%)	152 (28.5%)^*^	**<0.001**
4–6 months	10 (30.3%)	78 (17.8%)	18 (20.7%)	124 (23.2%)	0.107
7–11 months	4 (12.1%)	39 (8.9%)	12 (13.8%)	120 (22.5%)	**<0.001**
12–24 months	2 (6.1%)	21 (4.8%)	27 (31.0%)^*^	138 (25.8%)	**<0.001**
Duration of symptoms before admission (days)	14.0 (7.0, 15.0)	5.0 (4.0, 8.0)^**^	9.0 (5.0, 17.0)	10.0 (5.0, 15.0)	**0.001**
Duration of symptoms before admission group					
<7d *n* (%)	3 (9.1%)	285 (65.1%)^*^	30 (34.5%)^*^	200 (37.5%)^*^	**<0.001**
7–14d *n* (%)	12 (36.4%)	72 (16.4%)^*^	30 (34.5%)^**^	139 (26.0%)	**<0.001**
≥14d *n* (%)	17 (51.5%)	81 (18.5%)^*^	27 (31.0%)	195 (36.5%)	**<0.001**
Clinic presentation					
Cough	33 (100.0%)	430 (98.2%)	84 (96.6%)	529 (99.1%)	0.236
Dyspnea	2 (6.1%)	18 (4.1%)	0 (0.0%)^**^	54 (10.1%)	**<0.001**
Rhinorrhea	15 (45.5%)	195 (44.5%)	21 (24.1%)^**^	218 (40.8%)	**0.005**
Vomiting	12 (36.4%)	78 (17.8%)^**^	9 (10.3%)^*^	117 (21.9%)	**0.005**
Cyanosis *n* (%)	4 (12.1%)	12 (2.7%)^*^	0 (0.0%)^*^	36 (6.7%)	**<0.001**
O_2_ requirement [*n* (%)]	2 (6.1%)	153 (34.9%)^*^	12 (13.8%)	100 (18.7%)	**<0.001**
Crackles *n* (%)	14 (42.4%)	291 (66.4%)^*^	54 (62.1%)	292 (54.7%)	**<0.001**
Laboratory findings					
Leukocyte count (×109/L)	16.8 ± 6.7	9.0 ± 4.0^*^	9.5 ± 3.7^*^	11.3 ± 5.9^*^	**<0.001**
Leukocyte count >15 × 10^9^	19 (57.6%)	33 (7.5%)^*^	6 (6.9%)^*^	106 (19.9%)^*^	**<0.001**
Lymphocyte count (%)	42.5 ± 28.6	60.8 ± 13.4^*^	48.2 ± 18.4	53.3 ± 24.4	**0.006**
Neutrophil count (%)	21.4 ± 21.7	32.9 ± 44.1	44.4 ± 18.6^**^	40.0 ± 21.0	**<0.001**
Hospital stay (day)	9.0 (7.0, 12.0)	8.0 (7.0, 9.3)^**^	8.0 (7.0, 9.0)^**^	8.0 (7.0, 10.0)	**0.011**

*B. pertussis–only infection: detection of B. pertussis without coinfection with any other virus or MP; RSV-only: detection of RSV without coinfection with any other virus or B. pertussis; MP-only: detection of RSV without coinfection with any other virus or B. pertussis; other pathogens: pathogens excluding B. pertussis–only, RSV-only, and MP-only infection. Data are expressed as % of positive cases, mean (quartile), unless otherwise stated. p < 0.05 considered statistically significant, listed in bold text, and represents p values for comparisons across all groups. Asterisks indicate statistical significance (p < 0.05) in bivariate comparison (B. pertussis–only vs. RSV-only, MP-only, and other pathogens infection*.

** *p < 0.008*,

* *p < 0.05)*.

In unadjusted comparisons, among 120 patients with *B. pertussis* and MP infections excluding co-detection with other pathogen types, children with *B. pertussis*–only infection were younger than children with MP-only infection (median 3.9 vs. 5.8 months, respectively) (Table 2). Children with *B. pertussis*–only infection were significantly more likely to have dyspnea (6.1%), rhinorrhea (15%), vomiting (15%), cyanosis (4%), and leukocyte count >15 × 10^9^/L (57.6%) compared with those with MP-only infection (0.0, 24.1, 10.3, 0.0, and 6.9%, respectively; *p* < 0.05 for all comparisons). Children with *B. pertussis*–only infection had a higher number of leukocyte and higher percentage of lymphocyte compared with children with MP-only infection. Children with *B. pertussis*–only infection had a longer duration of hospital stay (median 9.0 days) than those with MP-only infection (median 8.0 days); however, no significant difference was observed in the duration of symptoms before admission.

### *B. pertussis* Infection and Results of Laboratory Examination in Different Age Groups

*B. pertussis–*positive patients were divided into three age groups to assess the difference among different age groups (Table 3). A total of 31 patients aged ≤3 months, 36 patients aged 4–11 months, and 11 patients aged ≥12 months were analyzed. Patients aged ≤3 months had a longer duration of hospital stay than others (*p* < 0.05). The common clinic characteristics among the 78 confirmed patients were paroxysmal cough 92.3% (72/78), whoops 15.5% (12/78), post-tussive vomiting 38.5% (30/78), and cyanosis 12.8% (10/78). Patients with cyanosis aged ≤3 months were more compared with older ones (*p* < 0.05); the others exhibited no difference among three age groups (*p* > 0.05). Patients aged ≤3 months requiring supplemental oxygen were more compared with older ones (*p* < 0.05). The gender ratio exhibited no significant difference among the three groups (*p* < 0.05). Coinfection among the three age groups was also compared, which showed no difference (*p* > 0.05). Although, patients aged ≥12 months had a higher number of leukocytes, and higher percentages of neutrophils and lymphocytes, no significant difference was observed among the three groups (*p* > 0.05).

**Table 3:** Clinical characteristics and results of laboratory examination among the different age groups with *B. pertussis* infection.

	**≤3 months (*n* = 31)**	**4-11 months (*n* = 36)**	**≥12 months (*n* = 11)**	***p*-value**
Clinical characteristics				
Hospital stay (day)	12.5 ± 6.69	9.91 ± 3.41	9 ± 1.56	0.049
Requirement for supplemental oxygen *n* (%)	10 (32.3%)	2 (5.6%)	1 (9.1%)	0.011
Paroxysmal cough *n* (%)	30 (96.8%)	33 (91.7%)	9 (81.8%)	0.237
Whoops *n* (%)	3 (9.7%)	9 (25.0%)	0 (0.0%)	0.076
Post-tussive vomiting *n* (%)	13 (41.9%)	12 (33.3%)	5 (45.5%)	0.694
Cyanosis *n* (%)	8 (25.8%)	2 (5.6%)	0 (0.0%)	0.030
Low oxygen saturation *n* (%)^a^	3 (9.7%)	2 (5.6%)	1 (9.1%)	0.728
Crackles *n* (%)	18 (58.1%)	27 (75.0%)	4 (36.5%)	0.051
Laboratory results				
Leukocyte count (×109/L)	15.83 ± 6.58	17.72 ± 8.39	14.25 ± 5.52	0.356
Lymphocyte (%)	38.57 ± 28.89	51.42 ± 26.63	35.77 ± 25.29	0.102
Neutrophil (%)	22.31 ± 22.73	20.15 ± 14.76	24.70 ± 18.31	0.723

### Clinical Features of *B. pertussis–*Only Infection Compared With Coinfection

Patients with *B. pertussis*–only infection were younger and had a high incidence of paroxysmal cough compared with patients with coinfection (*p* < 0.05). However, patients with coinfection had an increased demand for oxygen and showed more crackles in lungs (*p* < 0.05) (Table 4).

**Table 4:** Comparison between *B. pertussis*–only infection and coinfection.

	***B. pertussis*–only infection (*n* = 33)**	***B. pertussis* and virus coinfection (*n* = 45)**	***p*-value**
Gender (male) *n* (%)	21 (63.6%)	25 (55.6%)	0.473
Age ≤3 months *n* (%)	17 (51.5%)	12 (26.7%)	0.025
Vaccination	22 (66.7%)	27 (60.0%)	0.547
Oxygen *n* (%)	2 (6.1%)	11 (24.4%)	0.031
Paroxysmal cough *n* (%)	33 (100%)	39 (86.7%)	0.036
Whoops *n* (%)	6 (18.2%)	6 (13.3%)	0.558
Post-tussive vomiting *n* (%)	12 (36.4%)	18 (40%)	0.744
Cyanosis *n* (%)	4 (12.1%)	6 (13.3%)	0.874
Low oxygen saturation *n* (%)^a^	2 (6.1%)	4 (8.9%)	0.643
Crackles *n* (%)	14 (42.4%)	35 (77.8%)	0.001

*B. pertussis–only infection: detection of B. pertussis without coinfection with any other virus or MP*.

a *Low oxygen saturation is less than 92%*.

## Discussion

In recent years, an increasing incidence of pertussis has been reported in infants and young children (13). Several studies suggested that *B. pertussis* is a possible pathogen causing bronchiolitis in infants (8–10). Several investigators demonstrated that *B. pertussis* is a common pathogen of bronchiolitis (14, 15). A study conducted in Turkey identified 44 (25.6%) of the 172 infants with *B. pertussis* hospitalized for acute bronchiolitis (15). Another study showed *B. pertussis* involvement in 12 of 142 (8.5%) infants hospitalized for bronchiolitis in Finland (9). Yet other studies identified that *B. pertussis* was an uncommon pathogen in bronchiolitis (16). Pedro A. Piedra and his colleges found only four of 2,027 children admitted to the hospital as *B. pertussis* positive using PCR in the USA (10). Similarly, Walsh et al. found *B. Pertussis* infection in three of 488 patients (0.6%) in the emergency department (17). The present study found that 7.1% of infants and young children hospitalized with acute bronchiolitis had a positive *B. pertussis* test, which demonstrated that *B. pertussis* was a common pathogen in bronchiolitis. The variation in the prevalence of *B. pertussis* in children hospitalized with bronchiolitis among the studies might be due to the difference in climates, recruit criteria, and vaccination. According to the finding of this study, the peak incidence of *B. pertussis* infection was from May to July, with a total of 33 (50.15%) patients, which has not been reported before.

Studies reported that *B. pertussis* coinfection with other respiratory viruses was common in children hospitalized for bronchiolitis; the incidence rate was 36–67% (9, 14, 15, 18). However, the present study reported that 45 (57.7%) patients with *B. pertussis* were coinfected with other respiratory viruses, which was in agreement with previous studies. Some studies (6, 15, 19) suggested that coinfection with RSV was the most common in young children hospitalized for bronchiolitis with *B. pertussis* infection. However, in the present study, the most common coinfection respiratory viruses in children with *B. pertussis* hospitalized for bronchiolitis were HRV (35.9%), followed by MP (11.4%), and HBoV (7.7%); these differences in coinfection might be due to the heterogeneity of social demography and differences in study methods and periods.

Symptom duration before admission and hospital stay were more common in *B. pertussis–*only infection than in RSV-only infection (*p* < 0.05). It indicated that patients with *B. pertussis–*associated bronchiolitis often presented a longer course, which was consistent with the clinical symptoms of *B. pertussis* infection (20) and could help distinguish *B. pertussis–*associated acute bronchiolitis from other acute bronchiolitis etiologies. The present study compared *B. pertussis–*only infection with RSV-only infection in children with bronchiolitis. Children with *B. pertussis–*only infection requiring supplement oxygen were fewer than children with RSV-only infection, indicating that the former had a milder clinical presentation compared with the latter. This study also compared *B. pertussis–*only infection with MP*-*only infection and infections with other pathogens in children and revealed similar severity among these pathogens. This is a novel report explaining such associations. Several other studies (9, 14, 15, 18, 20–22) assessed the influence of *B. pertussis* on acute bronchiolitis, but they could not exclude the possibility of other respiratory pathogens contributing to the illness. In the present study, the leukocyte count was higher in patients with *B. pertussis–*only bronchiolitis infection than that in patients with RSV*-*only infection, MP*-*only infection, and infections with other pathogens (*p* < 0.008 for all comparisons), which could also help distinguish *B. pertussis–*associated acute bronchiolitis from other acute bronchiolitis etiologies. One study showed that the leukocyte count > 60 × 10^9^/L was associated with death in children with *B. pertussis* infection (23). Another study demonstrated that the leukocyte count > 100 × 10^9^/L was an independent risk factor of death in children with pertussis (24). However, no patent died of *B. pertussis* infection in the present study, which might be because the vast majority of infants and young children with mild-to-moderate bronchiolitis were considered, and severe bronchiolitis in the PICU setting was ignored.

Pertussis is a vaccine-preventable respiratory disease. *B. pertussis* could affect all individuals, but the highest morbidity and mortality rates were among newborns and unvaccinated or incompletely vaccinated young infants (21, 25, 26). In the present study, the morbidity of *B. pertussis* was common (31/78, 39.7%) in infants with bronchiolitis who had been unvaccinated (infants ≤3 months). The unvaccinated infants were associated with a longer hospital stay and more likely to require supplemental oxygen. Studies suggested that early identification and treatment of *B. pertussis* could shorten the duration of paroxysmal cough (27, 28), and antibiotics against pertussis could limit the severity of disease if started in the catarrhal phase (27, 29). In addition, several systematic reviews confirmed the safety and effectiveness of maternal pertussis vaccination during pregnancy (30–32). Therefore, it is important to early recognize and initiate treatment.

This study had potential limitations. It enrolled only inpatients hospitalized with *B. pertussis* infection, but more patients with *B. pertussis* infection were treated in the outpatient department. Therefore, patients with more severe symptoms might have been overrepresented, and the prevalence of *B. pe*rtussis in children with bronchiolitis-associated hospitalization might be affected.

In summary, *B. pertussis* is one of the pathogens in children with bronchiolitis, and coinfection of *B. pertussis* with other viruses is common in bronchiolitis. *B. pertussis* should be considered when patients hospitalized with bronchiolitis present a longer course and have an elevated leukocyte count. Patients with *B. pertussis–*associated bronchiolitis present a milder clinical presentation.

## Data Availability Statement

The raw data supporting the conclusions of this article will be made available by the authors, without undue reservation.

## Ethics Statement

The studies involving human participants were reviewed and approved by Ethics committees of Children's Hospital Soochow University (Approval No.: 2016026). Written informed consent to participate in this study was provided by the participants' legal guardian/next of kin.

## Author Contributions

WJ and SC wrote the main manuscript text. CH and YW designed the study and revised the manuscript. ZC and MW carried out the initial analyses. XS and JX performed the microbiological detection. AL and QX performed the data collection. All authors read and approved the final manuscript.

## Conflict of Interest

The authors declare that the research was conducted in the absence of any commercial or financial relationships that could be construed as a potential conflict of interest.

## Publisher's Note

All claims expressed in this article are solely those of the authors and do not necessarily represent those of their affiliated organizations, or those of the publisher, the editors and the reviewers. Any product that may be evaluated in this article, or claim that may be made by its manufacturer, is not guaranteed or endorsed by the publisher.
